# A New Technique Using Low Volumes: A New Technique to Assess the Molluscicidal Activity Using Low Volumes

**DOI:** 10.1155/2017/3673197

**Published:** 2017-08-29

**Authors:** José Augusto Albuquerque Santos, Viviane Paixão Cavalcante, Leonardo da Silva Rangel, João Claudio Vitória Atico Leite, Robson Xavier Faria

**Affiliations:** ^1^Laboratório de Avaliação e Promoção da Saúde Ambiental, Instituto Oswaldo Cruz, Fiocruz, Av. Brasil 4365, Manguinhos, 21045-900 Rio de Janeiro, RJ, Brazil; ^2^Laboratório de Toxoplasmose e outras Protozooses, Instituto Oswaldo Cruz, Fiocruz, Av. Brasil 4365, Manguinhos, 21045-900 Rio de Janeiro, RJ, Brazil

## Abstract

Schistosomiasis is a disease endemic in several states of Brazil. The population control of the transmitter mollusks is done with Bayluscide WP 70®, in the control programs. OMS preconize molluscicidal assays using Becker with 500 mL of final volume, restringing the number of natural products and synthetic drugs to be tested in function of high quantity of material necessary. A new technique to assess the toxic effects for* Biomphalaria* sp. is the purpose of this work, for developing adaptation for this aquatic organism, using a low volume of test solution in 24-well plates. We used* Biomphalaria glabrata* (10–12 mm size) in a static system, consisting of the following components: Becker containing 10 snails or 24-well plates where snails were individualized for a volume of 2 mL per well for 24 and 48 hours. For the assays, we added aqueous solutions of Bayluscide WP 70, at a concentration of 1–5 mg/L, distilled water, and 1% dimethyl sulfoxide. Data were evaluated using Kappa's coefficient, *Z* factor validation, and comparison study. This technique to assess the toxic effect has proven to be a useful tool to detect lethal and sublethal effects, which could be used as a new evaluation protocol.

## 1. Introduction

Schistosomiasis is a disease of acute and chronic character, endemic in several states of Brazil. The population control of transmitter mollusks is done with niclosamide, a molluscicide recommended by the World Health Organization for control programs [1–3].

The biological response should be combined with chemical analyses and with toxicological assessments that are commonly performed using acute toxicity tests because they are relatively simple to perform and produce fast results. However, environmental contamination in natural ecosystems often occurs at concentrations well below the lethal concentration, which may cause sublethal effects [4].

A promising field investigation was done with the latex of* Euphorbia splendens *var.* hislopii* for the control of schistosomiasis vector mollusk in restricted lentic habitats [5, 6].

Usually, the molluscicidal activity is measured using Becker with 10 snails. The habitual volume used to do the assay is Becker with 250 mL [7], but there is a description of assays using a volume of 500 mL with 10 mollusks [8]. In both situations, the quantity of substance used is extremely high for the volume used. In consequence, a large number of compounds derived from crude plant extracts, such as fractions, isolated and purified substances, and essentially synthetic substances, are neglected due to the amount required to develop biological tests with mollusks, because, in the protocol preconized for OMS, these compounds should be tested in a high final volume. Then, the plant extract or synthetic drug concentration necessary to reach the final doses will be elevated to cause a molluscicidal activity.

In this scenario, the purpose of this work was to develop a new technique to assess the toxic effects of synthetic substances, extracts, fractions, and compounds isolated from plants of* Biomphalaria* sp. using low volumes for aquatic organisms, with the use of disposable 24-well plates.

## 2. Materials and Methods

### 2.1. Snails

**Figure 1:**
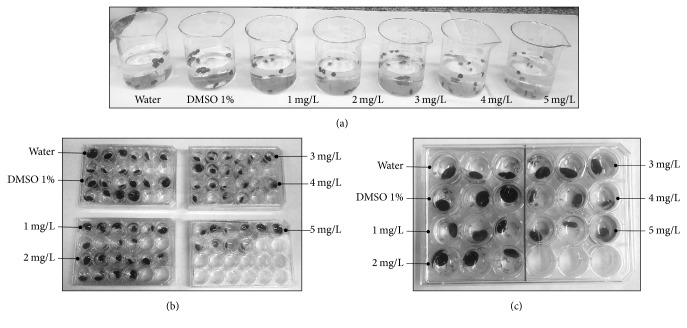
Molluscicidal evaluation models with niclosamide on* Biomphalaria glabrata* under laboratory conditions. (a) Becker of 1000 mL, containing the control groups, water, and 1% aqueous dimethylsulfoxide, 500 mL of aqueous solutions of niclosamide (1 mg/L, 2 mg/L, 3 mg/L, 4 mg/L, and 5 mg/L), and 10* Biomphalaria glabrata*, with a size between 10 and 12 mm in diameter by Becker, created and kept in the laboratory. (b) Methodology with 24-well plates, containing the control groups and the doses of niclosamide and 1 snail per well with 2 mL of solution. Exposure of 10 animals per dose as described in (a). (c) Methodology with 24-well plates, containing the control groups and the doses of niclosamide and 1 mollusk per well with 2 mL of solution as described above. Exposure of 3 animals per dose.

For tests with adult* Biomphalaria glabrata *(i.e., 1818 Mollusca, Gastropoda, and Planorbidae) snails, the vector of* Schistosoma mansoni*, a static system was used, consisting of the following components: Becker containing 10 snails (Figure 1(a)) and 24-well plates with cover and the test substances in solution (Figure 1(b)).

We used* Biomphalaria glabrata *(10–12 mm standard size) from Sumidouro, Rio de Janeiro, RJ, maintained in aquaria with dechlorinated water and fed with lettuce in the laboratory.

### 2.2. Period of Exposure and Aqueous Solution Preparation

The* Biomphalaria glabrata *mollusks were exposed for 24 and 48 hours at different doses of niclosamide, in triplicate. We used the doses of 1 mg/L, 2 mg/L, 3 mg/L, 4 mg/L, and 5 mg/L of Bayluscide WP 70, initially dissolved in DMSO (dimethylsulfoxide) (experimental group), distilled water (negative control group), and 1% DMSO solution (negative control group).

### 2.3. Tests with the Mollusk

The final volume for each dose was 500 mL with Becker containing 10 snails. In the case of the 24-well plate, each well contained a final volume of 2 mL with one snail. In a condition, we used 10 wells with 10 mollusks for treatment (1 mollusk per well) and another 3 wells with 3 mollusks for treatment (1 mollusk per well). The observations were performed by analyzing morphological and functional criteria.

Each snail was individualized in 24-well plates, and a volume of 2 mL of the dose to be tested was added per well for the experimental group or controls and covered with a lid.

For the assays,* B. glabrata* were added to aqueous solutions of niclosamide, prepared in the laboratory at doses of 1 mg/L, 2 mg/L, 3 mg/L, 4 mg/L, and 5 mg/L (experimental group), distilled water (negative control group), and 1% DMSO solution (negative control group). To assess the toxic effect with a small volume of solution, the total number of animals used in triplicate tests was 21 snails and that used in decaplicate tests was 30 snails. During this period, the 24-well plates containing the doses were kept at room temperature and the animals were not fed. Each test was conducted for periods of 24 h, 48 h, and 72 h exposure. The tests were realized at least on three distinct days. The percentage of dead snails using a total number of 9 or 30 snails was calculated estimating the niclosamide molluscicidal activity as 100%. Triplicate or decaplicate assays were repeated on three distinct days. All other values were normalized in function niclosamide result.

### 2.4. Characterization of the Snails' Death

The deaths of the animals during the tests were confirmed by the change in shell color, absence of muscle contractions, whether the cephalopodal mass was distended in a distinguished fashion, and the presence of the middle* Miliammina*.

### 2.5. Extracts

The staff of the Natural Products Technology Laboratory FFU (LTPN) at the Jurubatiba Sandbank National Park, in the municipality of Carapebus, RJ, collected aerial parts of the plants of concern:* Manilkara subsericea*. The herborization of the material was made by a botanist, Prof. Dr. Marcelo Guerra Santos, and exsiccated specimens were deposited at the Herbarium of the Teacher Training College (UERJ). The lab obtained authorization from SISBIO/ICMBio, code 51842449, to collect plants from Restinga Jurubatiba National Park.

In the LTPN, parts of stems and collected plants were dried in an oven at 40°C with forced ventilation for two days. Once dry, this material was triturated in a hammer mill and subjected to extraction by maceration or percolation using ethanol as a solvent. This was followed by filtration and concentration in a rotary evaporator, thereby obtaining the crude extract. Aliquots of the extracts thus obtained were further diluted in DMSO and then serial dilutions were made in distilled water for the experiments.

### 2.6. Ethics Approval and Consent to Participate (Ethics Statement)

On July 2, 2015, representatives of the Oswaldo Cruz Institute's Ethics Committee on Animal Use (CEUA-IOC) met and decided on the basis of the documents presented; the committee decided on the need to issue a CEUA license. The project presented to this committee does not meet the requirements of Law 11794 of 2008 regulating animal research in Brazil. Article 2 of this law applies to animals of the species classified as Filo Chordata, sub-Vertebrata, subject to environmental legislation. Specifically, the project refers to the use of* Biomphalaria* sp. (Filo Mollusca) in tests of potent molluscicidal agents.

### 2.7. Data Analysis

The mortality rate was normalized to the maximum value using Microsoft Excel, and the results were plotted using the GraphPad Prism version 3.0 (San Diego, CA, USA). Data were expressed as mean ± SD (standard deviation), as indicated in the text. One-way Analysis of Variance (ANOVA), followed by Tukey's test, tested the statistical significance of the differences between means. A bicaudal *p* < 0.05 was considered significant.

The LC_50_ values were calculated by graphics from drug concentration versus lethality percentage using a dispersion analysis, which provides an equation for calculating the LCs.

We compare the efficacy of the methodologies using the coefficient “Kappa” (*K*) of Cohen that measures the nonrandom proportional agreement [9] of results independently of the laboratorial methodology used. A *K* value of 0.5 indicates a moderate level of agreement between the techniques. A value of *K* > 0.80 represents an excellent proportional agreement and not random. For this calculation, we used the program Win Episcope 2.0®.

## 3. Results

**Figure 2:**
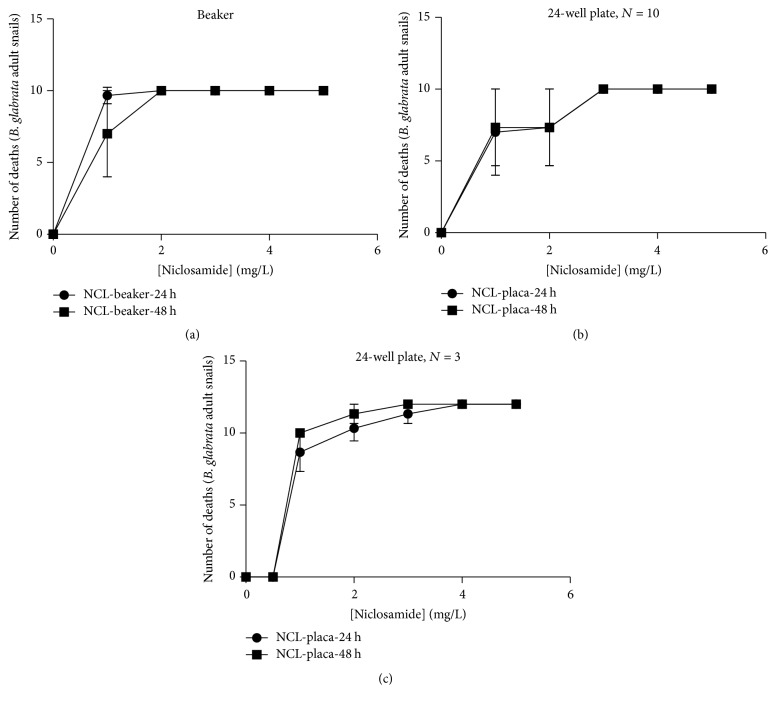
Niclosamide induced death of adult* B. glabrata* snails. (a) Dose-response graph representing niclosamide concentrations (24- or 48-hour stimulus) in the function of death of mollusks measured in the Becker methodology with 10 snails. (b) Dose-response graph representing niclosamide concentrations (24- or 48-hour stimulus) in the function of death of mollusks measured in the 24-well plate methodology with 10 snails. (c) Dose-response graph representing niclosamide concentrations (24- or 48-hour stimulus) in the function of death of mollusks measured in the 24-well plate methodology with 3 snails. The concentrations of the added blockers are given in the text. Experiments were performed for at least three independent days.

In Figure 2, we plotted niclosamide dose-response graphs against the number of dead mollusks treated for 24 and 48 hours. The linear regression analysis performed on the 24-well plate test with 10 animals, during a period of 24 and 48 hours, presented values of *R*^2^ = 0.5057 and 0.5081 and standard deviations of 3.155 and 3.089, respectively. The regression analysis in the tests was considered significant, with *p* value < 0.0009 in both periods.

In the methodology using 24-well plates with 3 snails (1 mollusk per well), the linear regression presented values of *R*^2^ = 0.7014 and 0.6665, with standard deviation of 0.7091 and 0.6711 for 24 and 48 hours, respectively. The regression analysis in the tests was considered significant, with *p* value < 0.0009 in both periods.

Additionally, in the Becker with 10 mollusks, the regression results were *R*^2^ = 0.4502 and 0.5032, with deviation of 2.917 and 3.034 for 24 and 48 hours, respectively. The regression analysis in the tests was considered significant, with *p* value < 0.0009 and *p* value < 0.001 in the periods.

**Figure 3:**
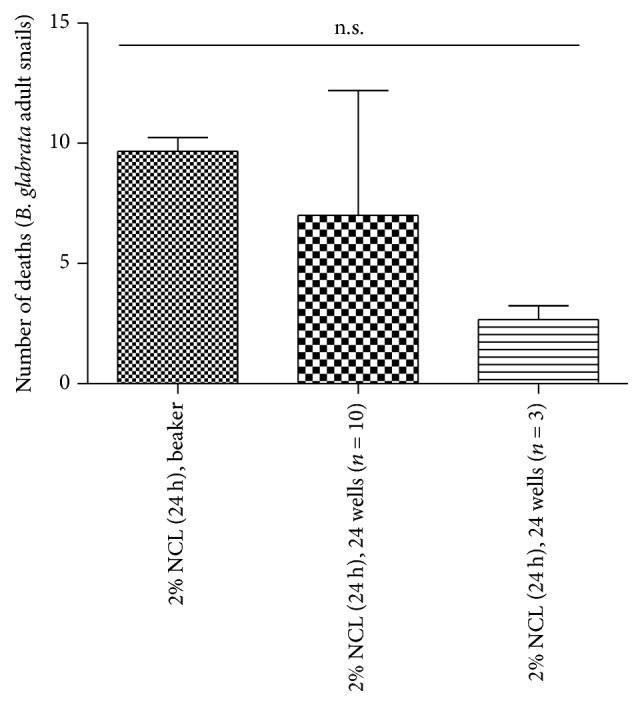
Comparison of the methodologies in relation to dose of 2% niclosamide against adult* B. glabrata* snails. Treatment for 24 hours with 2% niclosamide. These results are representative of three distinct days.

When we compared the results in the dose of 2% niclosamide among three methodologies for 24-hour of treatment, there was no statistical difference (Figure 3).

Regarding the results, we evaluated whether the optimized protocol reducing the volume and number of mollusks was comparable to the protocol with Becker or not. We compared the two protocols using Kappa's coefficient.  Table 1 represents the number of dead mollusks collected in a standard assay after 24 or 48 hours of treatment, on 3 distinct days, using Becker with niclosamide concentrations in a final volume of 500 mL and 10 snails. In another assay, we treated individually 10 mollusks in wells with a final volume of 2 mL, also on three different days. Both assays were recorded until 48 hours, because after this time all mollusks were dead at all concentrations and in all protocols used. The values in the columns were used to do the contingence table to calculate Kappa's coefficient.

The strength of concordance, according to Landis and Koch [10], shows that the results of 24 and 48 hours for 1 mg/L niclosamide exhibited a moderate correlation. The dose of 2 mg/L showed a considerable association and all other doses presented perfect concordance (Table 1).

In Table 2, we compared Becker's protocol with the 24-well protocol containing 3 mollusks in a final volume of 2 mL. Exceptionally, the concordance between these protocols was more satisfactory for all doses. The results of 1 mg/L in 24 and 48 hours showed that a *K* value greater than 0.5 indicates a moderate to excellent level of agreement between the techniques. In the dose of 2 mg/L after 24 hours also, it was shown that a *K* value greater than 0.5 indicates a moderate to excellent level of agreement between the techniques and after 48 hours it was almost perfect. All other doses in the times of 24 and 48 hours exhibited an almost perfect correlation (Table 2).

When we compared the 24-well plate assays containing 10 or 3 snails, the low dose tested showed acceptable correlation after 24 hours and moderate correlation after 48 hours. In the dose of 2 mg/L, there was considerable correlation after 24 hours, but moderate correlation after 48 hours. In accordance with all correlations tests, in the doses ranging from 3 to 5 mg/L, there was almost perfect correlation (Table 3).

We evaluated the validation of the optimization using 3 mollusks in the 24-well plate, when we measured the *Z* factor. This coefficient also can be used to estimate the quality of assays in high throughput screening (HTS), which made it possible to use this factor with volume and number of mollusks reduced (Table 4). Then, we used each dose of niclosamide as a positive control to observe the quality of the assay in all doses. The *Z* factor for 1 mg/L was 0.92, 0.67, and 0.73 for the Becker, 24 wells with 10 mollusks, and 24 wells with 3 snails assays, respectively. According to Zhang et al. [11], all assays exhibited excellent performance. All other doses after 24 or 48 hours presented a *Z* factor with a value considered excellent and comparable to Becker's assay (Table 5).

We compared also the assays in relation to other reliability parameters calculated. In this case, we evaluated the sensitivity, specificity, positive predictive value (PPV), negative predictive value (NPV), and prevalence according to Watson and Petrie (2010). In Table 6, we added the values referent to comparison between Becker and 24 wells with 10 mollusks treated for 24 hours. The sensitivity for all doses was almost 100%, and the specificity was about 50% for the doses of 1 and 2 mg/L, but the other doses reached maximal specificity. PPV, NPV, and prevalence exhibited values superior to 70% for all doses tested (Table 6).

In the results after 48 hours, there was sensitivity of 82.69% in the dose of 1 mg/L; all other doses were 100%. Specificity for 1 and 2 mg/L was 68 and 50%, respectively. However, this parameter increased at the doses of 3, 4, and 5 mg/L to 100%. PPV exhibited values above 80% for all doses as observed after 24 hours. NPV was 65.83% for the dose of 1 mg/L but reached 100% in all doses. Prevalence was augmenting in accordance with the dose concentration, 67.53% for 1 mg/L and 76.47% for 2 mg/L to 100% in the other doses (Table 7).

When we compared Becker's assay with 24-well plate with 3 snails, in general, the results were extremely similar to the plate with 10 snails. The sensitivity was almost 100% for all doses tested after 24 hours of treatment. The specificity was about 50% in the doses until 3 mg/L, but it augmented for 100% in the doses of 4 and 5 mg/L. PPV and NPV were higher than 80% for all doses and the prevalence only was below 90% at the dose of 1 mg/L (Table 8).

The results of 48 hours demonstrated sensitivity of 75% at the dose of 1 mg/L and 100% for all doses. The specificity was maximal for higher doses, 71.42 and 50% for doses of 1 mg/L and 2 mg/L, respectively. PPV values were above 80% for all doses and NPV was maximal for all doses except dose of 1 mg/L with 62.50%. Only the dose of 1 mg/L had prevalence of 66.66%; others were higher than 95% (Table 9).

We also compared the assays using 24-well plate with 10 mollusks and 3 snails. The results for 24 hours indicate sensitivity of 70% for 1 mg/L and values superior to 90% for all doses. The specificity was 76.47% at the dose of 1 mg/L, 83.33% for 2 mg/L, 50% for 3 mg/L, and 100% for all doses. PPV values were superior to 80% and NPV values were 59.09% and 55.55% for doses of 1 and 2 mg/L, respectively. In the higher doses, the NPV values were 100%. Prevalence in the dose of 1 mg/L and 2 mg/L was 63.82% and 75.51%; the other doses were above 95% (Table 10).

In the assays after 48 hours, sensitivity, specificity, and PPV were 90% higher for all doses except for 1 mg/L that presented sensitivity and specificity values of 77.77% and 78.57%, respectively. NPV values were maximal for doses of 3–5% and 55.89% and 52.94% for 1 and 2 mg/L. Prevalence values were higher than 70% for all doses tested (Table 11).

In relation to reliability, the doses of 3, 4, and 5 mg/L, in general, showed results almost maximal for 24 or 48 hours in all assays tested. The doses of 1 and 2 mg/L exhibited a higher fluctuation. The minor value observed for sensitivity was 70%; the specificity, in general, was about 50%. PPV and NPV values were always above 80%; however, NPV values for comparison between 24-well plate assays were reduced to values in turn of 50%. Prevalence values also were well satisfactory ranging from 63% to 95%. These results indicate a high correlation, reliability, and quality of the 24-well plate assays when compared to Becker's assays to measure the death of the snails.

**Figure 4:**
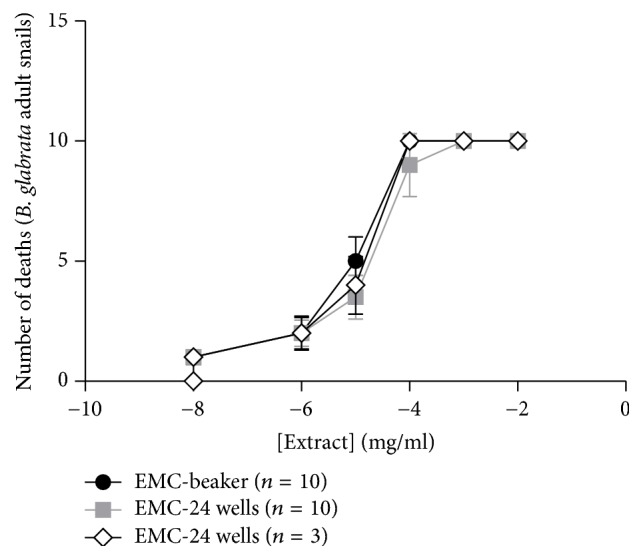
Dose-response effect of crude ethanol extract of stems of* Manilkara subsericea *against* Biomphalaria glabrata*. We treated the snails with crescent doses of the extract for 48 hours. These experiments were realized in three distinct days.

Based on these results, we compared the molluscicidal effect of crude ethanol extract of stems of* Manilkara subsericea* in the three methodologies (Figure 4). The IC_50_ values calculated for Becker's assay were 11.36 ± 3.87 *μ*g/mL and 18.2 ± 4.8 *μ*g/mL for 24 wells with 10 snails and 15 ± 2.6 *μ*g/mL for 24 wells with 3 snails. According to these values, the IC_50_ values calculated were similar between these mythologies, reinforcing the results with 24 wells and three snails.

## 4. Discussion

Our aim was to optimize a protocol to measure the molluscicidal action of plant extracts and essentially purified substances. This objective was based on the difficulty to discover new potential drugs against adult mollusks of* B. glabrata* in function of large volumes necessary to evaluate the molluscicidal action and consequently to reduce the quantity of material needed to perform the assay. This point also reflects the cost to develop a new drug and generally abolish the opportunity to test synthetic molecules.

The proposal of this paper was to diminish the volume used to dissolve the tested substances and decrease the number of mollusks used, and then we could test a large number of plant extracts, purified substances, or synthetic molecules against death of the snail.

According to WHO recommendations, the molluscicidal activity is evaluated using 10 mollusks exposed for 48 hours in a final volume of 500 mL [7]. Meanwhile, there are a large number of papers describing variations in these conditions and observing satisfactory results. There are descriptions using plastic terrariums with 9 cm diameter and 6 cm depth. The final volume was 5 mL with mollusks exposed through dermal contact directly with the extract [12]. Silva and collaborators [13] used plastic terrariums with capacity of 250 mL containing 5 adult mollusks exposed to a final volume of 8 mL sprayed with manual sprayer.

de Souza and colleagues also used terrariums; however, they pulverized 10 mollusks for terrariums with 20 mL of extract solutions [14].

In another paper, the authors used a Petri dish of 14 cm with 10 mollusks and a final volume of 100 mL to evaluate plant extract and Becker of 30 mL containing 10 mollusks and a final volume of 10 mL with purified substances [15].

There are papers using Becker with a maximal volume of 250 mL containing 10 mollusks for treatment [13, 16, 17], 500 mL with 10 mollusks [8, 18, 19], 1000 mL with 10 mollusks [20, 21], and in rare cases 2000 mL [22]. However, these papers, changing the final volume and number of mollusks, did not validate mathematically the efficiency of these protocols to measure molluscicidal activity in comparison to standard protocol.

We applied multiple doses ranging from 1 mg/L to 5 mg/L niclosamide for 24 or 48 hours. All results recorded by the three distinct methodologies were compared in relation to concordance. For this, we analyzed two-by-two methodologies using a contingence table to calculate Kappa's coefficient. We calculated a Kappa value to each dose tested, and then we observed possible discrepancies in accordance with the final dose used. The values obtained for doses ranging from 3 to 5 mg/L, in general, reached maximal correlation. In the doses of 1-2 mg/L, there was higher oscillation, and the strength of agreement to Becker's assay and 24-well plate with 10 mollusks was moderate at both doses after 24 hours of exposition. The agreement with the dose of 2 mg/L was good and that with 1 mg/L was moderate after 48 hours.

In the comparison between Becker and 24 wells with three snails, the concentration in both doses exhibited good strength of agreement after 24 hours. In 48 hours, the dose of 2 mg/L reached an almost perfect agreement and the dose of 1 mg/mL showed good correlation. All superior doses were practically perfect between these methodologies.

When we compared 24-well plates containing 10 snails for each treatment or 3 snails for each treatment, the results observed with respect to doses ranging from 3 to 5 mg/L were unchanged, when was compared with other correlations analyzed. However, there was a considerable reduction in the strength of agreement to doses of 1 and 2 mg/L. The dose of 2 mg/L exhibited a good strength after 24 hours, in contrast to the dose of 1 mg/L with a result close to *K* value of 0.5 indicating a moderate level. A *K* value of 0.5 indicated that a moderate level at 48 hours of exposition was moderated to both doses.

Based on these results, the assays in the concentrations ranging from 3 to 5 mg/L were comparable among all methodologies tested. In the doses of 1 and 2 mg/L, there was a higher variation. In relation to 24-well plate with 10 mollusks compared to Becker's assay, it is indicated to do assays with dose of 2 mg/L niclosamide. In the assays using 24-well plate with 3 mollusks compared with Becker's assay, all doses tested may be used as satisfactory, essentially after 48 hours of exposition. Then, we considered the methodology in 24-well plate with 3 mollusks as more efficient to evaluate molluscicidal activity.

Taking into consideration the better performance of methodology in 24-well plate containing 3 mollusks and its large potential to screen plant extracts and principally purified or synthetic molecules, we evaluated quality, reliability, and robustness properties.

Initially, we calculated the *Z*′ factor for all methodologies focusing on the methodology of 24-well plate with 3 snails. As represented in Table 5, all assays displayed ideal or excellent results, when these doses were considered as maximal value after 24 or 48 hours of treatment. Then, we measured reliability factors for these methodologies. Becker's assays for 24 hours reached 100% for sensitivity, specificity, PPV, NPV, and prevalence in the doses varying from 3 to 5 mg/L (Table 6). In the doses of 1 and 2 mg/L, the specificity was in turn 50% and the prevalence inferior to 80%; all other parameters were superior to 85%. After 48 hours, the dose of 1 mg/L showed the worst reliability results (Table 7). Other doses exhibited results similar to 24 hours. The 24-well plate containing 10 mollusks treated for 24 hours was similar to Becker's assays; however, the specificity was 50% also in the dose of 3 mg/L and the dose of 1 mg/L did not reach the maximal values; however, it reached a satisfactory value (Table 8). After the exposition of 48 hours, all doses were above 90% with the exception of 1 mg/L, where the sensitivity and specificity were above 70%, but the NPV and prevalence were 62, 50% and 66, 66%, respectively (Table 9). The assay using 24-well plate with 3 mollusks in 24 hours was over 90% for all parameters in the doses of 3–5 mg/L; only the specificity in the dose of 3 mg/L was 50%. The dose displayed reduced parameters of NPV and prevalence, but the specificity and sensitivity were reasonable (Table 10). For 48 hours, all other values considerably rose for almost maximal values; the unique exception was NPV values.

## 5. Conclusion

These results demonstrated 24-well plate as a good tool to optimize Becker's assays reducing the final volume and number of snails, and thus a large number of plant extracts and essentially synthetic and purified molecules may be tested against mollusk survival. Consequently, this result opens a new perspective to discover and develop a novel drug capable of controlling the population number of* B*.* glabrata *mollusks diminishing the risk of schistosomiasis transmission.

## Figures and Tables

**Table 1:** Comparison between two protocols (Becker and 24-well plate) using Kappa's coefficient. *N* = 10.

Niclosamide concentrations (mg/L)	Becker *N* = 10 24 h (number of dead snails)	24-well plate *N* = 10 24 h (number of dead snails)	Becker *N* = 10 48 h (number of dead snails)	24-well plate *N* = 10 48 h (number of dead snails)	Kappa coefficient 24 h	Kappa coefficient 48 h
1	29	21	21	22	0.589	0.501
2	30	22	30	22	0.604	0.707
3	30	30	30	30	1	1
4	30	30	30	30	1	1
5	30	30	30	30	1	1

**Table 2:** Comparison between two protocols (Becker and 24-well plate) using Kappa's coefficient. *N* = 3.

Niclosamide concentrations (mg/L)	Becker *N* = 10 24 h (number of dead snails)	24-well plate *N* = 3 24 h (number of dead snails)	Becker *N* = 10 48 h (number of dead snails)	24-well plate *N* = 3 48 h (number of dead snails)	Kappa coefficient 24 h	Kappa coefficient 48 h
1	29	5	21	6	0.661	0.795
2	30	7	30	8	0.792	0.965
3	30	8	30	9	0.943	1
4	30	9	30	9	1	1
5	30	9	30	9	1	1

**Table 3:** Comparison between two protocols (24-well plate, *n* = 10 with *n* = 3) using Kappa's coefficient. *N* = 3.

Niclosamide concentrations (mg/L)	24-well plate *N* = 10 24 h (number of dead snails)	24-well plate *N* = 3 24 h (number of dead snails)	24-well plate *N* = 10 48 h (number of dead snails)	24-well plate *N* = 3 48 h (number of dead snails)	Kappa coefficient 24 h	Kappa coefficient 48 h
1	21	5	22	6	0.389	0.45
2	22	7	22	8	0.738	0.556
3	30	8	30	9	0.971	1
4	30	9	30	9	1	1
5	30	9	30	9	1	1

**Table 4:** A simple interpretation of screening assay quality by the value or the *Z* factor.

*Z* factor	Interpretation
1.0	Ideal *Z* factors can never exceed 1
Between 0.5 and 1.0	An excellent assay. Note that if *σ*_*p*_ = *σ*_*n*_, 0.5 is equivalent to separation of *12* standard deviations between *μ*_*p*_ and *μ*_*n*_
Between 0 and 0.5	A marginal assay
Less than 0	There is too much overlap between the positive and negative controls for the assay to be useful

**Table 5:** Validation of the optimization using 3 mollusks in a 24-well plate, with *Z* factor.

Niclosamide concentrations (mg/L)	Becker (*N* = 10) *Z* factor 24 h	Becker (*N* = 10) *Z* factor 48 h	24-well plate (*N* = 10) *Z* factor 24 h	24-well plate (*N* = 10) *Z* factor 48 h	24-well plate (*N* = 3) *Z* factor 24 h	24-well plate (*N* = 10) *Z* factor 48 h
1	0.92	0.67	0.67	0.92	0.73	1
2	1	1	0.70	1	0.85	0.89
3	1	1	1	1	0.89	1
4	1	1	1	1	1	1
5	1	1	1	1	1	1

**Table 6:** Assessment of sensitivity, specificity, positive predictive value (PPV), negative predictive value (NPV), and prevalence (24 hours).

Niclosamide concentrations (mg/L)	Becker (*n* = 10) × 24-well plate (*n* = 10) 24 h (% sensitivity)	Becker (*n* = 10) × 24-well plate (*n* = 10) 24 h (% specificity)	Becker (*n* = 10) × 24-well plate (*n* = 10) 24 h (% PPV)	Becker (*n* = 10) × 24-well plate (*n* = 10) 24 h (% NPV)	Becker (*n* = 10) × 24-well plate (*n* = 10) 24 h (% prevalence)
1	98.03	52.63	84.74	90.90	72.85
2	100	50	86.66	100	76.47
3	100	100	100	100	100
4	100	100	100	100	100
5	100	100	100	100	100

**Table 7:** Assessment of sensitivity, specificity, positive predictive value (PPV), negative predictive value (NPV), and prevalence (48 hours).

Niclosamide concentrations (mg/L)	Becker (*n* = 10) × 24-well plate (*n* = 10) 48 h (% sensitivity)	Becker (*n* = 10) × 24-well plate (*n* = 10) 48 h (% specificity)	Becker (*n* = 10) × 24-well plate (*n* = 10) 48 h (% PPV)	Becker (*n* = 10) × 24-well plate (*n* = 10) 48 h (% NPV)	Becker (*n* = 10) × 24-well plate (*n* = 10) 48 h (% prevalence)
1	82.69	68	84.31	65.38	67.53
2	100	50	86.66	100	76.47
3	100	100	100	100	100
4	100	100	100	100	100
5	100	100	100	100	100

**Table 8:** Comparative study on the Becker test with 24-well plates with 3 snails.

Niclosamide concentrations (mg/L)	Becker (*n* = 10) × 24-well plate (*n* = 3) 24 h (% sensitivity)	Becker (*n* = 10) × 24-well plate (*n* = 3) 24 h (% specificity)	Becker (*n* = 10) × 24-well plate (*n* = 3) 24 h (% PPV)	Becker (*n* = 10) × 24-well plate (*n* = 3) 24 h (% NPV)	Becker (*n* = 10) × 24-well plate (*n* = 3) 24 h (% prevalence)
1	96.66	55.55	87.87	83.33	75
2	100	50	94.87	100	90.24
3	100	50	97.43	100	95
4	100	100	100	100	100
5	100	100	100	100	100

**Table 9:** Comparative study on the Becker test with 24-well plates with 3 snails.

Niclosamide concentrations (mg/L)	Becker (*n* = 10) × 24-well plate (*n* = 3) 48 h (% sensitivity)	Becker (*n* = 10) × 24-well plate (*n* = 3) 48 h (% specificity)	Becker (*n* = 10) × 24-well plate (*n* = 3) 48 h (% PPV)	Becker (*n* = 10) × 24-well plate (*n* = 3) 48 h (% NPV)	Becker (*n* = 10) × 24-well plate (*n* = 3) 48 h (% prevalence)
1	75	71.42	81.81	62.50	66.66
2	100	50	94.87	100	95
3	100	100	100	100	100
4	100	100	100	100	100
5	100	100	100	100	100

**Table 10:** Comparative study on the 24-well plates test with 10 snails and 3 snails.

Niclosamide concentrations (mg/L)	24-well plate (*n* = 10) × 24-well plate (*n* = 3) 24 h (% sensitivity)	24-well plate (*n* = 10) × 24-well plate (*n* = 3) 24 h (% specificity)	24-well plate (*n* = 10) × 24-well plate (*n* = 3) 24 h (% PPV)	24-well plate (*n* = 10) × 24-well plate (*n* = 3) 24 h (% NPV)	24-well plate (*n* = 10) × 24-well plate (*n* = 3) 24 h (% prevalence)
1	70	76.47	84	59.09	63.82
2	93.54	83.33	93.54	55.55	75.51
3	97.43	50	97.43	100	95
4	100	100	100	100	100
5	100	100	100	100	100

**Table 11:** Comparative study in the 24-hour test in 24-well plates, with 10 snails and 3 snails.

Niclosamide concentrations (mg/L)	24-well plate (*n* = 10) × 24-wells plate (*n* = 3) 48 h (% sensitivity)	24-well plate (*n* = 10) × 24-well plate (*n* = 3) 48 h (% specificity)	24-well plate (*n* = 10) × 24-well plate (*n* = 3) 48 h (% PPV)	24-well plate (*n* = 10) × 24-well plate (*n* = 3) 48 h (% NPV)	24-well plate (*n* = 10) × 24-well plate (*n* = 3) 48 h (% prevalence)
1	77.77	78.57	90.32	57.89	72
2	96.77	90	96.77	52.94	77.55
3	100	100	100	100	100
4	100	100	100	100	100
5	100	100	100	100	100
